# Unveiling Vaginal Fibrosis: A Novel Murine Model Using Bleomycin and Epithelial Disruption

**DOI:** 10.4236/ojog.2025.153033

**Published:** 2025-03-19

**Authors:** Jennifer M. McCracken, Gisele A. Calderon, Felipe Rivas, Dorothea Erxleben, Taylor Moseley, Lishore A. Kumar, Daniel E. Kennedy, Swathi Balaji, Adam Hall, Julie C. E. Hakim

**Affiliations:** 1 Division of Pediatric and Adolescent Gynecology, Department of Obstetrics and Gynecology, Baylor College of Medicine, Houston, Texas, USA; 2 School of Biomedical Engineering and Sciences, Wake Forest University School of Medicine, Winston-Salem, North Carolina, USA; 3 Division of Pediatric Surgery, Department of Surgery, Texas Children’s Hospital, Houston, Texas, USA

**Keywords:** Vagina, Fibrosis, Wound Healing, Collagen, Hyaluronan, Epithelial Disruption

## Abstract

Vaginal fibrosis induced by cancer therapy continues to take a physical and psychoemotional burden. To advance the efforts to generate a reproducible and cost-effective animal model, we tested the effect of repeated bleomycin instillations with mucosal layer disruption on induction of vaginal fibrosis. Tissue samples collected at various time points were analyzed for fibrosis-related gene expression changes and collagen content. Low (1.5 U/kg) and high-dose (2.5 U/kg) bleomycin instillations alone did not induce fibrosis, but when high-dose bleomycin was combined with epithelial disruption, increased profibrotic gene expression and trichrome staining were observed. To evaluate spatial and temporal changes in the ECM structure and gene expression, tissue samples were collected at 1 day, 3 weeks, and 6 weeks after bleomycin and epithelial disruption. Data analyses revealed a significant decrease in matrix metabolizing genes and an increase in pro-fibrotic genes and inhibitors of matrix metabolizing genes in the bleomycin plus epithelial disruption group at 3 weeks. Elevated levels of the profibrotic genes *Acta2*, *Col1a1*, and *Col3a* were exclusively detected in this group at 3 weeks, and trichrome staining confirmed increased collagen content after 3 weeks. Both average hyaluronan (HA) size and mass distribution were increased 3 weeks after bleomycin plus epithelial disruption with return to baseline by 6 weeks. Epithelial disruption combined with bleomycin induces murine vaginal fibrosis within three weeks, characterized by increased collagen synthesis. The vaginal tissue fully recovers within six weeks, elucidating the regenerative capacity of the vagina.

## 1. Introduction

The vagina can sustain injury and immense tensional forces, such as those experienced during childbirth via vaginal delivery, yet it still has the remarkable potential for regenerative healing [1]. Under different wound healing conditions, such as reconstruction to correct congenital birth defects or after radiation exposure, the vagina has the potential to develop fibrosis and scar formation, causing significant pain and psychologic burden [2]. However, the specific cellular and molecular mechanisms that determine whether wound repair follows a regenerative or fibrotic pathway remain unknown. Considering the high burden of fibrotic vaginal wound complications, seen in up to 73% of post-surgical vaginal wound beds [3], research in this area is vital for improving global women’s health outcomes. A considerable number of girls and women who experience vaginal fibrosis, regardless of the underlying cause, are likely to develop some degree of vaginal stenosis or lumen narrowing. This may necessitate surgical intervention or ongoing vaginal dilatation for the remainder of their lives [4]. Therefore, to improve the quality of life for patients affected by vaginal fibrosis, we seek to identify the mechanisms during the vaginal repair process that contribute to uncontrolled, disordered healing to develop techniques and therapies aimed at prevention and early targeted intervention.

We previously established a surgical murine vaginal wound model, using a 1 mm full thickness punch biopsy which healed regeneratively, or without fibrosis, 72 hours after injury [5]. Given our previous work and the imperative need to create a vaginal injury model leading to fibrotic outcomes, our primary objectives of this project were to develop, validate, and characterize a model of murine vaginal fibrosis. In this work, we use repeated bleomycin vaginal instillations combined with traumatic disruption of the mucosal layer using bristle curettage to induce murine vaginal fibrosis.

Bleomycin is a clinical chemotherapeutic agent and bleomycin instillation is widely used to induce experimental fibrosis in multiple organ systems such as skin and lung [6] [7] where it has been shown to increase collagen deposition and hydroxyproline levels, cardinal features of organ scarring [7] [8]. Bleomycin exposure to the lung and specifically in the case of laryngotracheal stenosis induction, bleomycin coated wire brushes were used to cause both physical and chemical injury which in turn induced scar formation [9] [10]. Though this technique has not been previously used to induce vaginal fibrosis, the vaginal mucosa bears a striking resemblance to other mucosal tissues that have been studied previously, suggesting that this may be a viable method for inducing vaginal fibrosis.

## 2. Materials and Methods

### 2.1. Animal Care and Use

C57BL/6J female mice between 6 – 7 weeks old were purchased from the Center for Comparative Medicine at Baylor College of Medicine (BCM) and were socially housed under a 12 h/12h light/dark cycle with free access to standard food and water. All animal research was approved by the Institutional Animal Care and Use Committee (IACUC) of BCM, AN-7475, and conducted in accordance with the NIH Guide for the Care and Use of Laboratory Animals.

### 2.2. Vaginal Injury

Bleomycin sulfate (B8416-15U, Sigma Aldrich, St. Louis, MO) was reconstituted with sterile normal saline. Mice were weighed prior to each exposure and bleomycin doses were adjusted according to animal weight to achieve weight-corrected volumes of either low dose (1.5 U/kg) or high dose (2.5 U/kg) bleomycin vaginal instillations. Normal saline intravaginal instillations were used as control. Three experimental conditions included: low dose bleomycin, high dose bleomycin, and epithelial disruption followed by high dose bleomycin. Epithelial disruption was done by inserting a wire brush (0.079” diameter) intravaginally until it met resistance and rotating clockwise 6 times. Mice were sedated using isoflurane at a rate of 0.5 L/min. Eloxiject (Meloxicam, 5mg/mL, Henry Schein NDC 11695-6925-1) was given to each mouse at a dose of 5mg/kg prior to sedation. Normal saline or bleomycin was instilled intravaginally (with or without prior epithelial disruption) using a p100 pipette tip. Mice were kept sedated, with tail held suspended for 5 minutes to allow absorption, followed by applying an aquaphor healing ointment “plug” across vaginal opening. They were then transferred to clean caging and allowed to recover. This was repeated 5 times over the course of 10 days (i.e., every other day for 10 days). Mice were euthanized 1 day, 3 weeks, or 6 weeks after the last installation. Vaginal tissue was then collected by dissection similar to previously described methods [11] and either placed in 10% formalin (n = 7 – 8) or snap frozen for future analysis (n = 7 – 8 for gene expression, n = 3 for HA analysis).

### 2.3. Histology and Immunohistochemistry

Tissue placed in formalin at time of collection was dehydrated through a series of graded ethanol and embedded in paraffin (FFPE). Sections in the size of 5μm were then made for staining. To evaluate tissue fibrosis and collagen content, sections were stained using Gomori’s Trichrome stain (Leica, Buffalo Grove, IL) according to manufacturer’s directions using Leica Autostainer. Images were taken at 20X using Leica DMi8 microscope with a Leica DFC4500 camera and Leica Application Suite X (LAS-X). ImageJ was used to determine the total area of submucosal tissue and amount of collagen content (blue staining) in the area using the color masking plug in as described previously [8] [9]. Settings were as follows: Hue: 124 – 194, Saturation: 0 – 255, Brightness: 0 – 255. Data is reported as % positive area, and 3 images per mouse were averaged. Hydroxyproline was detected using a hydroxyproline detection kit (MAK008, Sigma Aldrich) per manufacturer’s instructions. Immunohistochemistry was used to localize total leukocytes and macrophages in the vaginal submucosa as described previously [3]. Briefly, FFPE sections were rehydrated, and the PT Link antigen retrieval system (Agilent, Santa Clara, CA) and low target retrieval solution (Agilent) were used. The Dako Autostainer Link 48 (Agilent) and appropriate Agilent reagents were used for blocking (S2003), antibody dilution (S0809), secondary antibody (K4009), and 3,3’-diaminobenzidine to visualize (DAB, K3468, Agilent). Hematoxylin (K8008) was used as a counterstain. CD45 (ab10558, 1:2000, AbCam) primary antibody was used. A Leica DMi8 microscope with Leica DFC4500 camera and LAS-X software was used to take 20X images (n = 3/mouse). ImageJ was used to determine the submucosal area in each image and to count the number of positive cells to calculate the number of positive cells/submucosal area.

### 2.4. Gene Expression

Vaginal tissue was flash frozen in liquid nitrogen at time of collection and stored at 80°C until RNA was isolated using a PureLink RNA Minikit (ThermoFisher) with an added DNA elimination step according to manufacturer’s directions. RNA was pooled so that there were equal amounts of RNA in each group per time point and each mouse in the group had equal contributions to the pooled sample. RNA was reverse transcribed using RT2 First Strand according to manufacturer’s instructions (Qiagen, Germantown, MD). RT2 Profiler PCR Array for Mouse Fibrosis (PAMM-120Z, Qiagen) and Qiagen GeneGlobe Analysis was used to evaluate fibrosis between normal saline and experimental groups at each time point. Standard qPCR was used to validate a selection of genes from the array. In brief, 220ng of RNA was reverse transcribed using High-Capacity RNA-to-cDNA kit (Fisher Scientific). Power SYBR green and Bio-Rad CFX thermocycler was used. Samples were done in duplicate, and data was analyzed using the ΔΔCt method and expressed as fold change over saline control at each time point. BioRad PrimePCR SYBR green assay (Cat 10025636) was used. Primers used include: *Acta2* (F: GACGCTGGATCCGATAGAACAACG, R: CACCATCTCCAGAGTCCAGCACAAAT), *Col1a1* (BioRad Cat#10025636), *Col3* (F: CTGTAACATGGAAACTGGGGAAA, R: CCATAGCTGAACTGAAAACCACC) and Rsp20 (F: GCTGGAGAAGGTTTGTGCG, R: AGTGATTCTCAAAGTCTTGGTAGGC).

### 2.5. Hyaluronan (HA) Detection

HA was localized as described previously [12] using a protocol adapted from the Cleveland Clinic’s Program for Excellence in Glycosciences. FFPE tissue sections were rehydrated, equilibrated in PBS, and blocked using avidin and biotin blocking kit (Vector Lab, Burlingame, CA). Normal goat serum was used as a blocking agent and slides were incubated in biotinylated HA binding protein (HABP, 1:500, CalBiochem, Burlington, MA) for one hour. The Avidin/Biotin complex (Vector Labs) was used to amplify the signal that was then detected with DAB (Vector Labs). Hematoxylin was used as a counterstain. A Leica DMi8 microscope with Leica DFC4500 camera and Las-X software was used to take 20X images (n = 3 per mouse). ImageJ was used to measure the area of the submucosa and the percent positive area over a threshold that was kept constant for each image. HA detection was performed by ELISA using a HA-specific ELISA (K-1200, Echelon Biosciences, Salt Lake City, UT).

### 2.6. HA Isolation from Murine Vaginal Tissue

HA isolation was performed by biomagnetic precipitation following protocols reported previously with minor modification [13]. Dynabeads^™^ M-280 streptavidincoated superparamagnetic beads (Ref. 11206D, Thermo Fisher Scientific) were first washed according to the manufacturer’s directions and then incubated (2 hours at room temperature under gentle agitation) in 1X PBS with biotinylated versican G1 domain (bVG1, Ref. G-HA02, Echelon Biosciences) at a ratio of 1 μg bVG1 to 100 μg beads. After washing and resuspension in 1X PBS to a concentration of 10 mg/mL, bVG1-conjugated beads were aliquoted into 150 μL volumes. Target tissue was diced and treated with a broad-spectrum protease (proteinase K, Ref. AM2548, Invitrogen, Waltham, MA) following the manufacturer’s directions until no solid material was apparent in the solution. Samples were placed on a heating block at 95°C for 10 min to deactivate protease. The digest was then mixed with an equal volume of phenol:chloroform:isoamyl alcohol, 25:24:1 (Ref. 327111000, Thermo Fisher Scientific, Waltham, MA) and transferred to a phase-lock gel tube (Ref. 2302830, QuantaBio, Beverly, MA) where it was centrifuged (13,000x rpm for 20 min at 20°C) to partition the mixture into an upper aqueous phase containing HA, nucleic acids, and other polysaccharides and a lower organic phase containing digestion proteins and other debris. The process was repeated twice with pure chloroform (Ref. AC423555000, Thermo Fisher Scientific) to remove residual phenol. The resulting solution was added to an aliquot of bVG1 beads and incubated at room temperature for at least 2 h for HA capture. Beads were pulled down magnetically from solution and then washed to counter non-specific binding. To elute HA, the beads were incubated in a high salt buffer (6M LiCl, 10 mM Tris, 1 mM EDTA, pH 8.0) for 1 h, after which they were again pulled out of suspension by magnetic field and the supernatant was collected for solid-state nanopore (SSNP) analysis.

### 2.7. HA Size Analysis by Solid-State Nanopore Analysis (SSNP)

SSNPs devices (silicon chips supporting a 30 nm thick silicon nitride membrane with a single pore of diameter 7 – 11 nm) were produced commercially by conventional silicon processing techniques (Norcada, Edmonton, Canada). To perform HA analysis [14], a chip was rinsed thoroughly with ethanol and water, dried with filtered air, treated with air plasma (30 W, Harrick Plasma, Ithaca, NY) for 2 min per side, and placed into a custom flow cell fabricated by 3D printing (Carbon, Redwood City, CA). Measurement buffer [15] (6M LiCl, 10 mM Tris, 1 mM EDTA, pH 8.0) was introduced into independent reservoirs on either side of the SSNP chip and Ag/AgCl electrodes were used to connect them to an Axopatch 200B patch-clamp amplifier (Molecular Devices, San Jose, CA). Isolated HA (in measurement buffer from the elution process) was then introduced to one side of the SSNP and measurements were performed by applying 200 – 300 mV to the opposite side and measuring current at a rate of 200 kHz using a 100 kHz four-pole Bessel filter. Data were collected with a custom LabVIEW program (National Instruments, Austin, TX) through which an additional 5 kHz low-pass filter was applied. Molecular translocations were signified by temporary reductions in the measured current (events), identified using an amplitude threshold of 5σ compared to baseline noise and inclusive only of events with durations in the range of 25 μs - 2.5 ms. The area of each event was mapped to a molecular weight using a calibration curve [16] produced with a comparable SSNP. Each data set consisted of a minimum of 500 individual events.

### 2.8. Statistical Analysis

Data was graphed and analyzed using GraphPad Prism Version 10.4.1. Data sets were tested for statistical outliers before being subjected to significance testing. Since our interest was in what effect vaginal injury had upon fibrotic hallmarks at each individual time point, a t-test was used to compare control and bleomycin + epithelial disruption at each single time point. As the number of mice per group were not evenly matched, t-tests were unpaired. An * was used to designate significance defined as p < 0.05. Q-test was used to remove any outlier at 95% confidence.

## 3. Results

### 3.1. Bleomycin and Epithelial Disruption in the Vagina Induce Pro-Fibrotic Gene Expression

**Figure 1.**
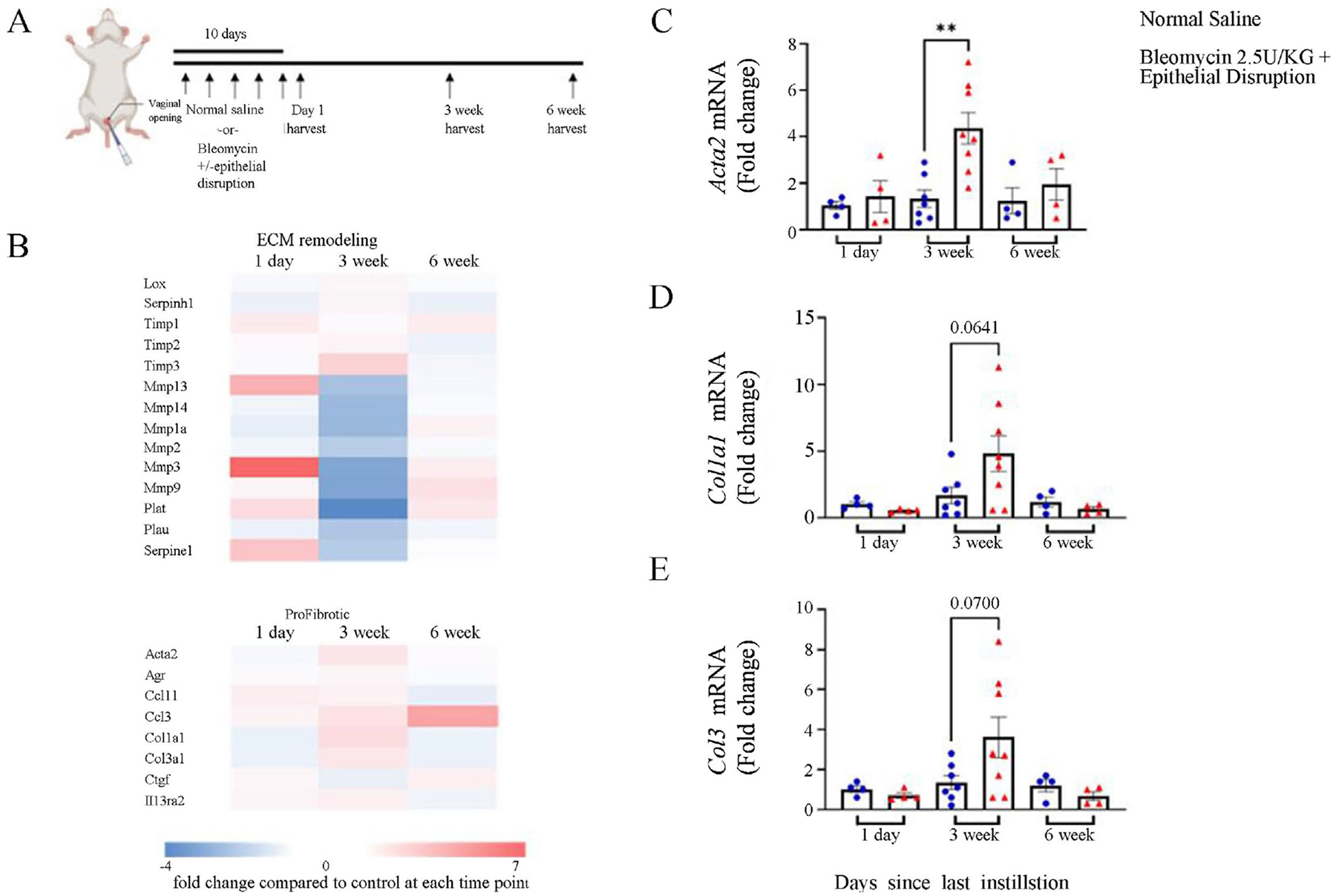
Gene expression analysis during vaginal fibrosis. (A) Schematic of model used for vaginal fibrosis studies. (B) Heat map of expressed genes associated with ECM remodeling and fibrosis. (C)-(E) qPCR validation of gene array results for *Acta2*, *Cot1a1*, and *Cot3*, respectively, at 3 weeks. For C-E, at 1 day, normal saline, n = 4, bleomycin plus ED, n = 4; at 3weeks, normal saline, n = 7, bleomycin plus ED, n = 8; at 6 weeks, normal saline, n = 4, bleomycin plus ED. n = 4. p < 0.01.

Bleomycin is a cellular toxin utilized to induce fibrosis in multiple organ systems in rodent models. Lung fibrosis has been induced using both a low dose bleomycin (1.5 U/kg) and high dose bleomycin (2.5 U/kg) [16] [17]. Similarly, laryngotracheal fibrosis has been induced using bleomycin coated wire brushes which disrupt the epithelial layer [9] [18]. Therefore, to induce vaginal fibrosis, we instilled a low dose (1.5 U/kg) and high dose (2.5 U/kg) of bleomycin intravaginally. In addition, we also included a high dose bleomycin instillation combined with wire brush epithelial disruption prior to instillation. To evaluate the progression of vaginal fibrosis, we harvested tissue at 1 day, 3 weeks, and 6 weeks after repeated bleomycin exposure plus epithelial disruption (Figure 1(A)). Neither low nor high dose bleomycin alone induced vaginal fibrosis evaluated by gene array and trichrome staining (data not shown). However, treatment with high dose bleomycin plus epithelial disruption resulted in down-regulation of genes encoding ECM metabolizing enzymes, particularly those involved in degradation, while genes associated with fibrosis and inhibitors of ECM metabolizing genes were up-regulated after 3 weeks (Figure 1(B)). We did not observe any consistent trends at 1 day or 6 weeks after bleomycin plus epithelial disruption compared to saline control at the corresponding time point. This was confirmed by qPCR (Figures 1(C)–(E)). We quantitated expression of three pro-fibrotic genes, *Acta2*, *Col1a1*, and *Col3a*, and found that all genes were elevated in the high-dose bleomycin plus epithelial disruption group compared to saline control at 3 weeks after the last instillation. This same difference was not observed at 1 day or 6 weeks post-installation.

### 3.2. Fibrotic Wound Resolution Is Accelerated in Vaginal Tissue

**Figure 2.**
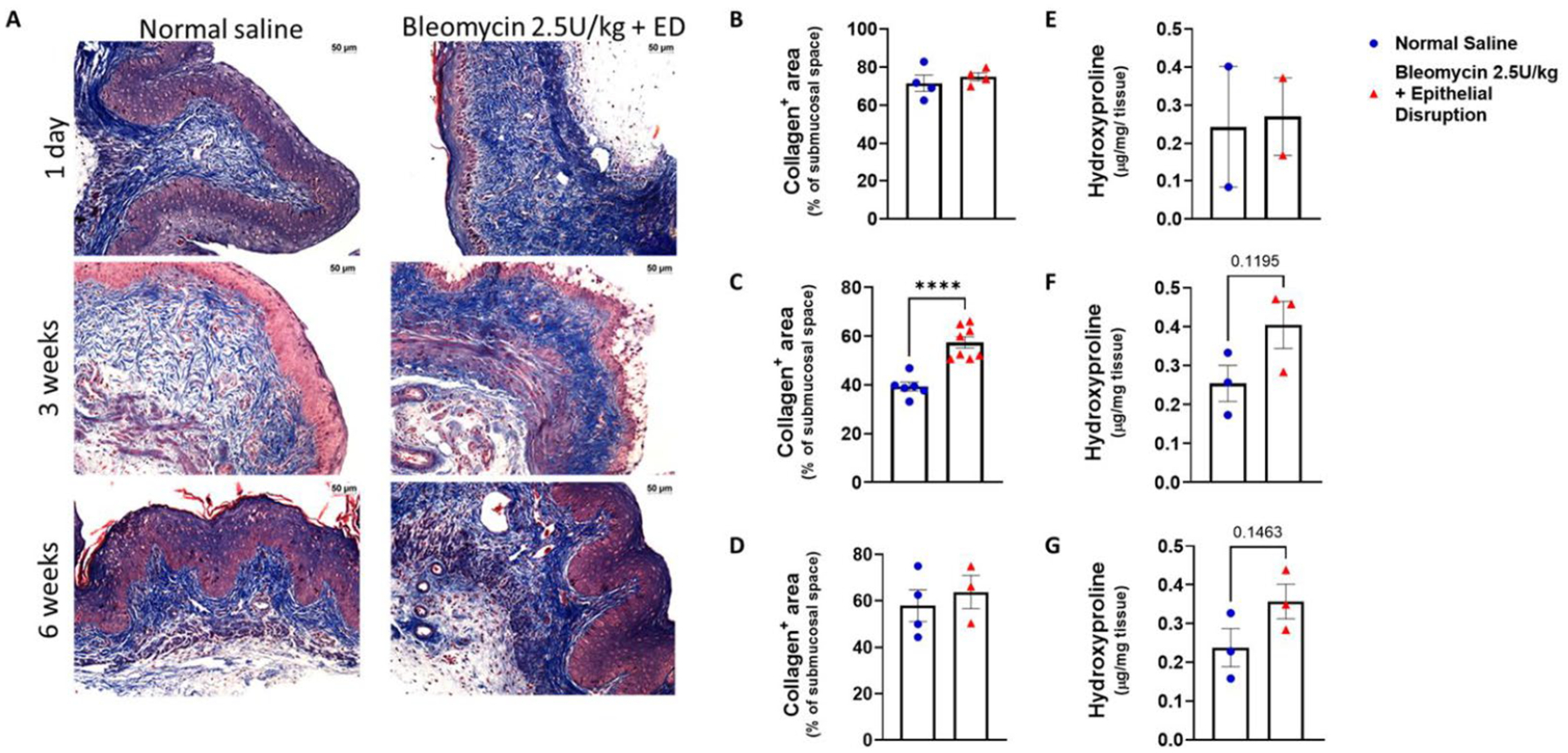
Collagen synthesis kinetics during vaginal tissue fibrosis. (A) Representative images of trichrome stained sections of vaginal tissue. Scale bars are 50 μm. (B)-(D) Collagen abundance represented as percentage of submucosal space of tissue. For (B)-(D), at 1 day, normal saline, n = 4, bleomycin plus ED, n = 4; at 3 weeks, normal saline, n = 6, bleomycin plus ED, n = 8; at 6 weeks, n = 4, bleomycin plus ED, n = 3. E-G. Hydroxyproline detection represented as concentration in vaginal tissue. For (E)-(G), at 1 day, normal saline, n = 2, bleomycin plus ED, n = 2; at 3 weeks, normal saline, n = 3, bleomycin plus plus ED, n = 3; at 6 weeks, normal saline, n = 3, bleomycin plus ED, n = 3. ***, p < 0.0001.

To confirm ECM remodeling we analyzed alterations in ECM protein expression by trichrome staining. There was increased collagen content in vaginal tissue exposed to bleomycin combined with epithelial disruption compared to saline control after 3 weeks of healing (Figure 2(A)). We observed no changes at 1 day or 6 weeks after the last vaginal instillation between our experimental mice and control mice (Figures 2(B)–(D)). A concomitant increased trend in hydroxyproline, an integral component of de novo synthesized collagen fibers, was also detected in vaginal tissues from the bleomycin plus epithelial disruption group at 3 weeks (Figures 2(E)–(G)) In conjunction with the gene expression data, these findings provide an insight into the time course of wound healing and fibrosis in our model, where fibrosis is not established within 24 hours of the final instillation of bleomycin and epithelial disruption. However, by 3 weeks, the presence of fibrosis is evident, and by 6 weeks after the last instillation, fibrosis has mostly resolved.

**Figure 3.**
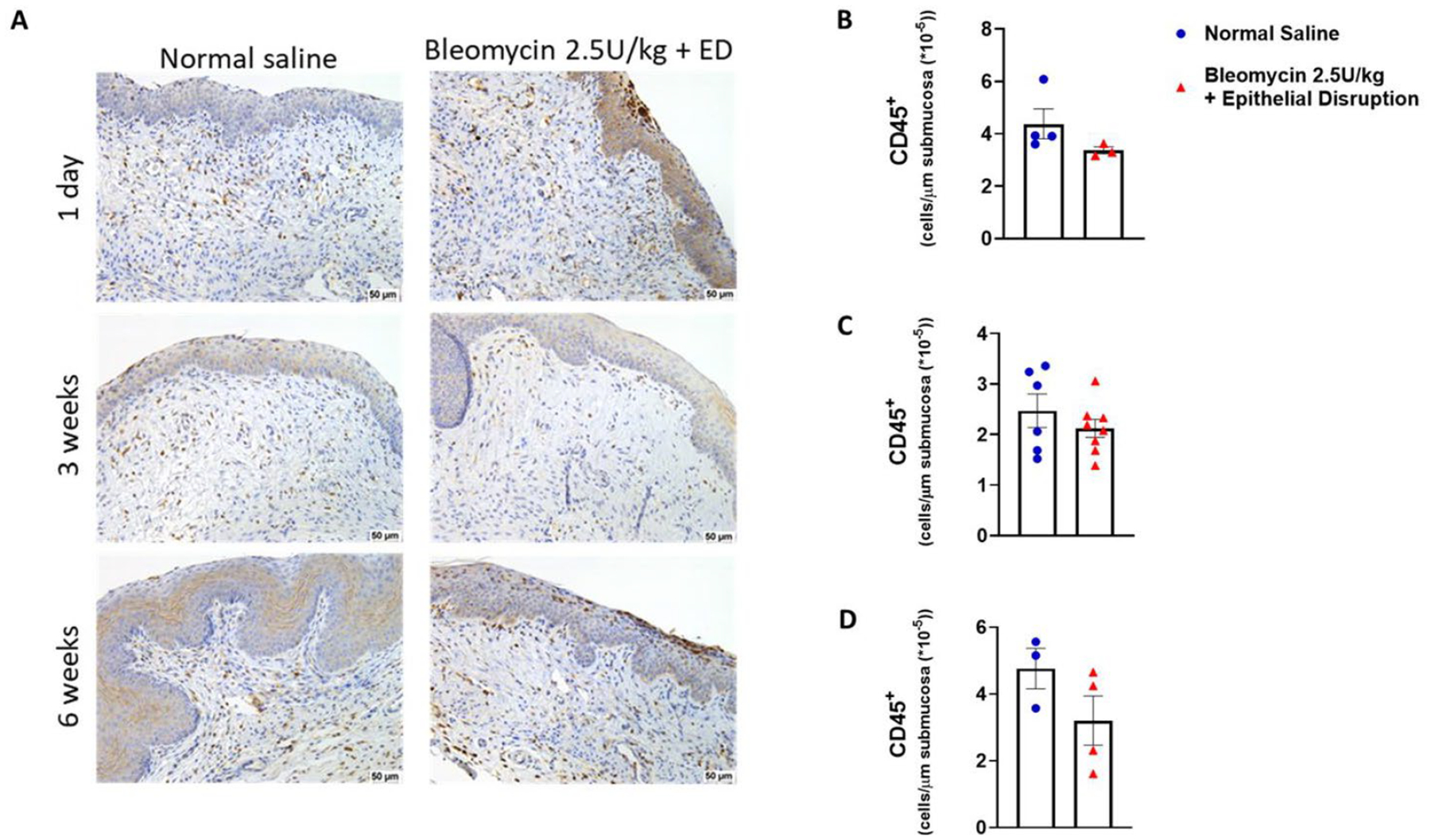
Immune cell recruitment during vaginal fibrosis. (A) Representative images of vaginal tissue sections stained for CD45 are shown. Scale bars are 50 μm. (B)-(D) Absolute numbers of immune cells are shown as CD45^+^ cells in submucosa. (B) At 1 day, normal saline, n = 4, bleomycin plus ED, n = 3; at 3 weeks, normal saline, n = 6, bleomycin plus ED, n = 8; at 6 weeks, normal saline, n = 3, bleomycin plus ED, n = 4.

We conducted immunohistochemical analysis to investigate vaginal inflammatory cell burden following bleomycin and epithelial disruption injuries. To determine the degree of inflammation in vaginal tissue after treatment, we quantitated the total number of leukocytes (CD45^+^) in tissue sections of control and bleomycin plus epithelial disruption mice at day 1, 3 weeks, and 6 weeks post last instillation (Figure 3(A)). Despite vaginal injuries, no difference in the total leukocyte abundance was observed at any time point (Figure 3(B)).

### 3.3. Vaginal HA Kinetics Are Impaired during Fibrosis Resolution

**Figure 4.**
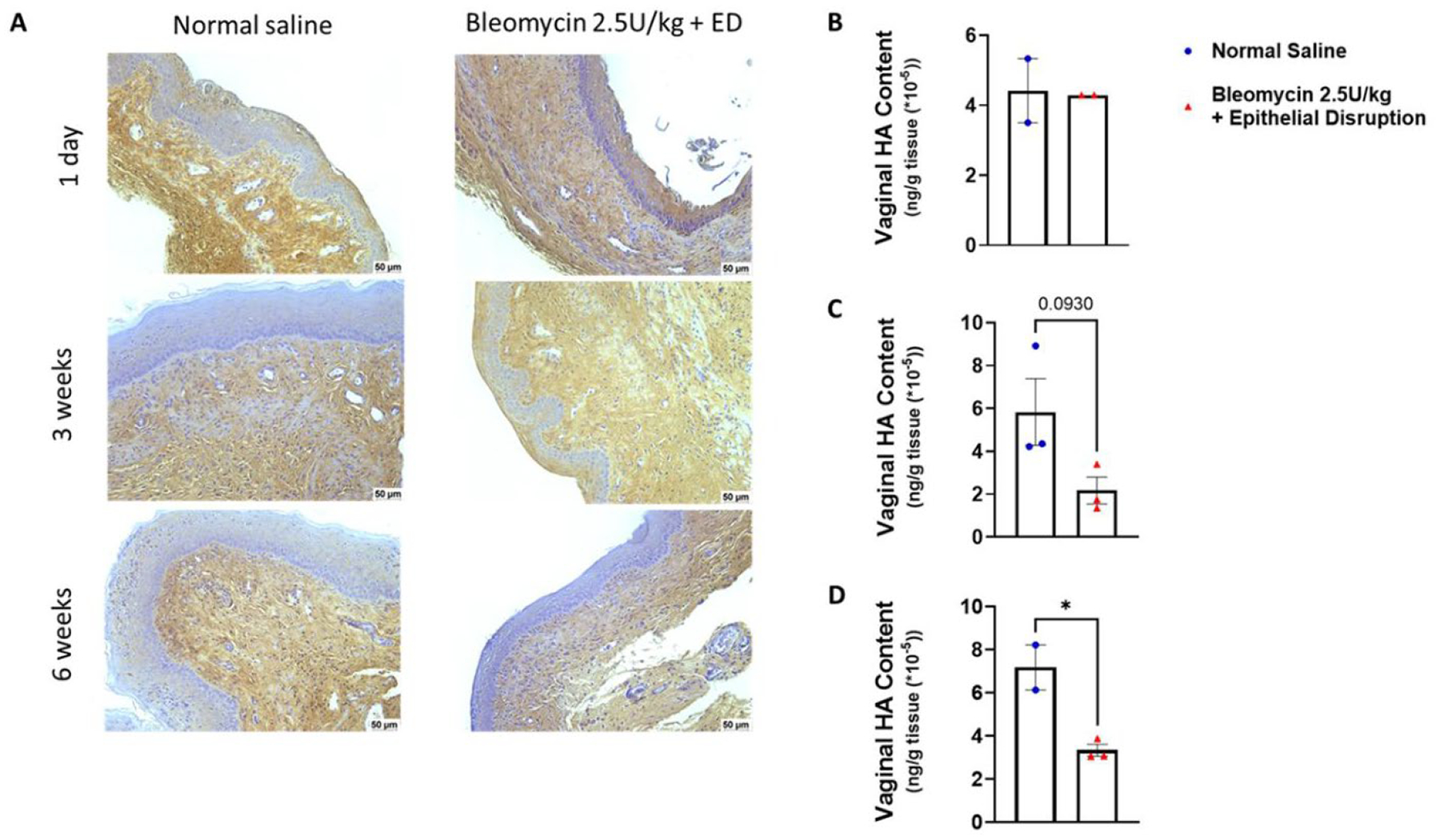
Changes in hyaluronan levels during vaginal fibrosis. (A) HA content determined by IHC HABP-staining of vaginal tissue sections. Scale bars are 50 μm. (B)-(D) Vaginal HA content is shown as concentration in whole fibrotic vaginal tissue. At 1 day, normal saline, n = 2, bleomycin plus ED, n = 2; at 3 weeks, normal saline, n = 3, bleomycin plus ED. N = 3; at 6 weeks. Normal saline, n = 2, bleomycin plus ED, n = 3. *, p < 0.05.

The vaginal ECM also contains abundant amounts of HA. To evaluate vaginal HA synthesis in response to bleomycin plus epithelial disruption and the development of fibrosis and subsequent resolution, we quantitated and characterized the flux of HA molecules within the vagina. Using HABP to localize HA expression in the wound sections, there was increased submucosal vaginal HA 3 weeks post bleomycin instillation and epithelial disruption (Figures 4(A)–(D)). However, using an ELISA, a trend toward decreased total vaginal tissue HA at 3 weeks was observed (Figure 4(C)), with a significant decrease at 6 weeks post-installation (Figure 4(D)).

**Figure 5.**
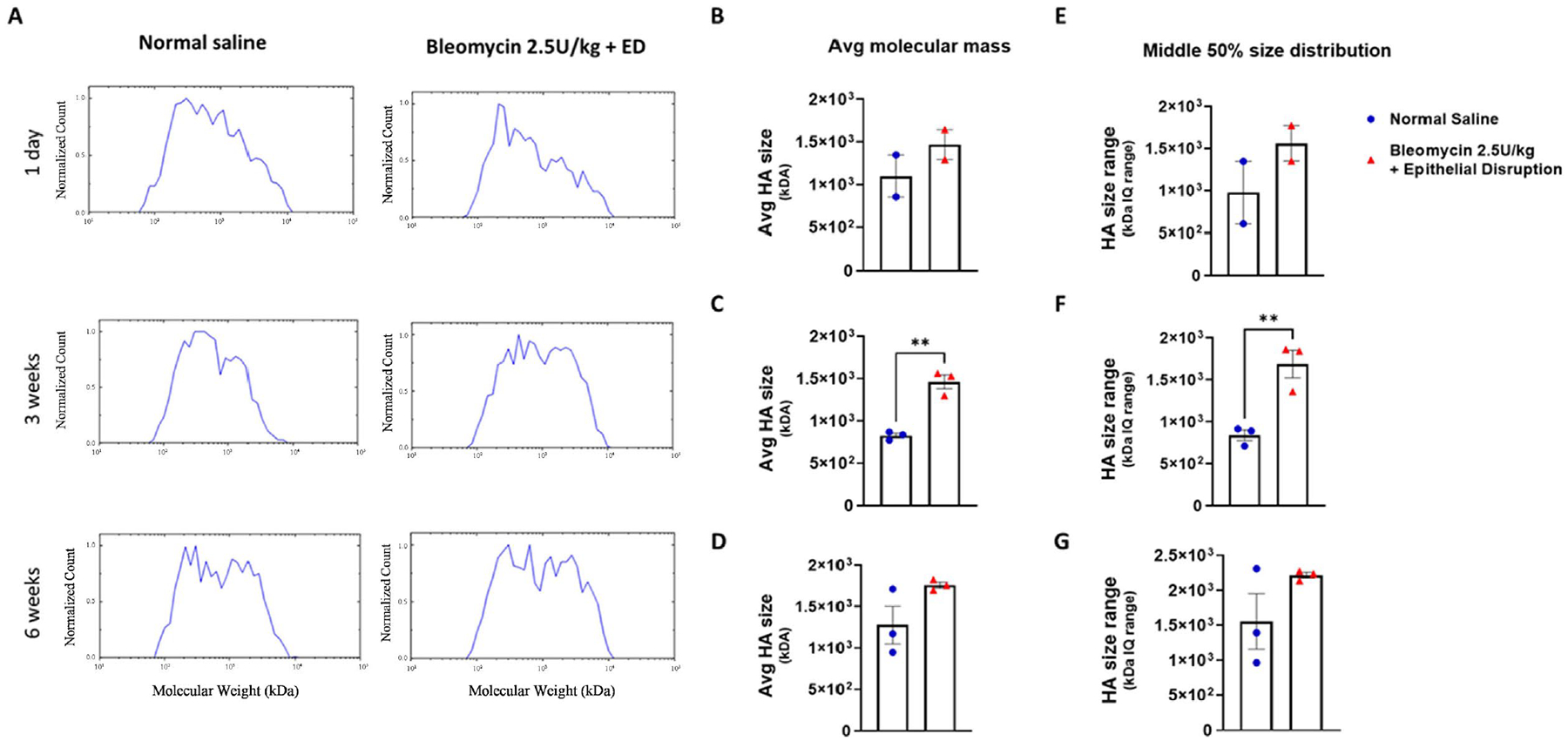
Determination of changes in hyaluronan molecular mass in fibrotic vaginal tissue. (A) Quantitation of molecular mass of HA is shown as the normalized count at each time point. (B)-(D) Average molecular mass of HA is shown at 1 day, normal saline, n = 2, bleomycin plus ED, n = 2; at 3 weeks, normal saline, n = 3, bleomycin plus ED, n = 3; at 6 weeks, normal saline, n = 3, bleomycin plus ED, n = 3. (E)-(G) HA size range is represented as the middle 50% size distribution at 1 day, normal saline, n = 2, bleomycin plus ED, n = 2; at 3 weeks, normal saline, n = 3, bleomycin plus ED, n = 3; at 6 weeks. Normal saline, n = 3, bleomycin plus ED, n = 3. **p < 0.01.

Since HA signaling is closely related to its molecular size, using HA isolated from the vaginas, we quantitated HA molecular mass by solid-state nanopore analysis. The average HA molecular mass was greater than that in mice 3 weeks after bleomycin plus epithelial disruption compared to controls (Figures 5(A)–(D)). To understand the size distribution of vaginal HA size, we evaluated the size range of the middle 50% of reads from the solid-state nanopore analysis. Compared to controls, in bleomycin plus epithelial disruption mice there was an increase in this size range 3 weeks post-injury (Figures 5(E)–(G)). No difference was found in either average molecular weight size or size distribution at 1 day or 6 weeks post bleomycin plus epithelial disruption. Collectively, our results demonstrate altered submucosal HA localization, and a reduction in vaginal HA levels following the onset of fibrosis, which remains at 6 weeks. Moreover, despite the total HA differences, there is a higher molecular weight of HA and a more diverse range of HA sizes within vaginal tissue when fibrosis is established at 3 weeks. These observations suggest an irregular HA turnover that persists even after fibrosis has been resolved.

## 4. Discussion

In this work, we have established and characterized a novel, reproducible, and site specific murine vaginal fibrosis model. Moreover, we have demonstrated the time course of vaginal wound healing from injury to fibrotic state, and subsequent resolution. This novel murine model eliminates many of the issues of previous murine vaginal injury models including small injury site, accelerated murine vaginal wound healing kinetics, and difficulty in creating reproducible surgical injuries leading to fibrosis while recapitulating clinical relevance using a gynecologic curettage technique [3]. This murine model will allow the development of novel preventions and treatments for vaginal fibrosis, a debilitating condition which plagues millions of women worldwide. Additionally, this model will serve as a tool to study the cellular and molecular mechanisms governing the spectrum of mucosal wound healing dynamics using the vagina as an archetype of these responses to injury.

With this model, we have also characterized several important molecular, cellular, and tissue alterations across the vaginal wound healing spectrum from fibrosis to regeneration. First, we clarified the genetic markers for vaginal states characterized by fibrosis and regeneration. In line with other tissues, our investigation revealed elevations in genes that promote fibrosis such as *Acta2*, *Col1a1*, and *Col3*, as well as changes in genes that regulate the breakdown and renewal of extracellular matrix (ECM) components, including matrix metalloproteases and inhibitors of matrix metalloproteases. These findings support the notion that the process of fibrosis involves a coordinated increase in ECM collagen production and a decrease in ECM degradation. The gene changes corresponded with vaginal fibrosis 3 weeks after injury and was confirmed both histologically and molecularly. We also demonstrate the need for epithelial disruption combined with chemical injury to be vital in inducing vaginal fibrosis, as chemical injury alone did not result in vaginal fibrosis.

Importantly, this model also contributes to the paradigm that the ECM is not a passive scaffold for wounds but actively participates in progression of both regeneration and fibrosis. It is possible that the vaginal ECM can repair itself following some degree of harm, such as with the use of bleomycin alone. However, once a certain level of damage is surpassed, the transcriptional responses that lead to fibrosis are triggered, requiring intervention. Furthermore, our findings demonstrate that fibrosis has been fully resolved within six weeks of injury, showing the murine vagina possesses an impressive capability to restore its equilibrium and regain homeostasis.

Inflammation often proceeds tissue injury to repair the tissue. Chronic inflammation has long been thought to be a contributing factor for fibrosis [19]. However, recent work has suggested that macrophage-mediated inflammation is necessary to counteract the exacerbated fibrosis [20]. The vaginal mucosa has sustained homeostatic leukocyte population necessary to protect the tissue from potential infection [21]. After inducing vaginal injury through repeated exposure to bleomycin in conjunction with epithelial disruptions, we observed that the total leukocyte populations remained relatively constant throughout the course of injury and regeneration, with no significant changes. Notably, even six weeks after injury, the leukocyte population remained slightly lower compared to control mice, despite the excess collagen in vaginal tissue having been resolved.

HA is one of the predominant ECM components in vaginal tissue [22]. This highly bioactive glycosaminoglycan is distributed widely in vaginal stroma and surrounding epithelial cells [23]–[25]. An increase of tissue HA during injury is a hallmark [26]. Differences in HA synthesis, accumulation, and degradation patterns can lead to aberrant wound healing [27]. The effect HA has on healing is largely dependent upon its molecular weight. Low molecular weight HA (LMW-HA) promotes a pro-inflammatory environment, while high molecular weight HA (HMW-HA) dampens pro-inflammatory effects [28]–[30]. The balance of LMW-HA to HMW-HA can also modulate inflammation during wound healing. The wound repair process leading either to fibrosis or regeneration can be described as a disordered or coordinated HA balance both in size and amount [31]. Regenerative tissue, such as fetal skin, has a high abundance of HMW-HA and tissues prone to scaring, including aged skin, have lower amounts of HMW-HA [27] [32]. Although HA is a key player in driving wound healing and fibrosis in other tissues, its specific role in mediating mucosal vaginal tissue responses to injury remains uncharacterized. As such, we have begun to characterize the flux of HA molecules to the vaginal wound bed by total amount and molecular mass. Total HA amounts decreased during collagen accumulation. However, the average molecular mass was highest in the vaginal tissue at 3 weeks when fibrosis was present and remained elevated at 6 weeks, despite the resolution of fibrosis. Additionally, when we consider the size range of HA, it was greater than at the time of vaginal fibrosis. Thus, we have demonstrated that HA turnover is altered during vaginal fibrosis progression, and re-establishing homeostasis of HA lags that of collagen in the injury-repair process.

Our study addresses the clinical need for a relatively low-cost, reproducible model of vaginal fibrosis. Globally, women’s rates of years lost to disability in high- and low-middle-income countries have risen to 3.94% and 5.35%, respectively, and vulvovaginal atrophic conditions tend to increase with age [33] [34]. Given the morphological changes in vaginal tissue of patients following radiotherapy, our model provides a viable option for investigating the underlying mechanisms of vaginal fibrosis [35]. Our findings indicate that vaginal fibrosis can be induced by epithelial disruption in conjunction with bleomycin vaginal instillation, leading to a noticeable increase in collagen synthesis and decrease in ECM degradation. This ultimately resulted in fibrosis within three weeks of the injury regimen. Additionally, we observed changes in other ECM components, including HA, in this injury model. Our findings revealed that the murine vagina possesses impressive regenerative abilities, able to fully recover within six weeks. These results provide a valuable platform for further investigation of the underlying mechanisms of vaginal fibrosis, as well as potential preventive and treatment strategies for the many women who suffer from this condition.

One of the limitations of this study is that the estrus cycles in the mice were not in sync. Furthermore, the signaling effects of estrogen on this injury model were not studied. Fundamentally, estrogen is known to modulate inflammation and angiogenic during dermal and vaginal wound healing [3]. Additionally, though bleomycin is a toxic anti-cancer agent known to cause fibrosis in other mucosal organs [9], we recognize its mechanism of action leading to fibrosis is likely different than surgically related or radiation induced vaginal fibrosis. Lastly, we recognize the potential small sample size used for HA analysis, and further studies to increase power would likely reduce the likelihood of statistical errors.

Overall, understanding the fundamental mechanisms that govern how the vaginal mucosa heals, both pathologically and regeneratively, will allow new insights into the design of personalized therapies to improve patient outcomes. With a novel mouse model of bleomycin with epithelial disruption, we can more robustly define the markers of pro-fibrotic phenotypes in mucosal tissues. Additionally, due to the ability of the murine vagina to resolve the established fibrosis, this model at different time points can be used to understand both the progression and resolution of fibrosis. Future directions include elucidating the interactions between tissue stretch and estrogen regulation of cellular processes that underpin the spectrum of vaginal wound repair. Since estrogen and HA are commonly used in vaginal therapy to treat and prevent fibrosis, our model provides the capability to extend the therapeutic repertoire in testing alternatives. Whether pathologic states of disordered wound healing could be susceptible to regulation by estrogen and biomechanical stretch and shift towards a regenerative phenotype sooner may promise solutions for women undergoing curettage or vaginal surgical repairs, and vaginal radiation therapies.

## Abbreviations and Acronyms

DAB: 3,3’-diaminobenzidine
DMAB: 4-(dimethylamino) benzaldehyde
ECM: Extra cellular matrix
ED: Epithelial disruption
FFPE: Paraffin
HA: Hyaluronan
HABP: Hyaluronan binding protein
HD: High dose
HMW-HA: High molecular weight hyaluronan
LAS-X: Leica Application Suite X
LD: Low dose
LMW-HA: Low molecular weight hyaluronan
SSNP: Solid state nanopore
